# *p53* polymorphisms associated with mutations in and loss of heterozygosity of the *p53* gene in male oral squamous cell carcinomas in Taiwan

**DOI:** 10.1038/sj.bjc.6602271

**Published:** 2004-12-07

**Authors:** L-L Hsieh, T-H Huang, I-H Chen, C-T Liao, H-M Wang, C-H Lai, S-H Liou, J T-C Chang, A-J Cheng

**Affiliations:** 1 Department of Public Health, Chang Gung University, Tao-Yuan, Taiwan; 2 Graduate Institute of Basic Medical Science, Chang Gung University, Tao-Yuan, Taiwan; 3 Department of Otolaryngology Oncology, Head and Neck Surgery, Chang Gung Memorial Hospital, Tao-Yuan, Taiwan; 4 Division of Hematology/Oncology, Department of Internal Medicine, Chang Gung Memorial Hospital, Tao-Yuan, Taiwan; 5 Department of Public Health, National Defense Medical Center, Taipei, Taiwan; 6 Department of Radiation Oncology, Chang Gung Memorial Hospital, Tao-Yuan, Taiwan; 7 Department of Medical Technology, Chang Gung University, Tao-Yuan, Taiwan; 8 Taipei CGMH Head and Neck Oncology Group, Tao-Yuan, Taiwan

**Keywords:** oral squamous cell carcinoma, *p53*, polymorphism, mutation, loss of heterozygosity

## Abstract

The present study was designed to examine whether different *p53* haplotypes of exon 4–intron 3–intron 6 affect the frequency of mutations and loss of heterozygosity (LOH) of the *p53* gene in male oral squamous cell carcinomas (OSCCs) in Taiwan. We found that individuals without two Pro-W-G alleles had significantly higher frequency of *p53* mutations than those with two Pro-W-G alleles (odds ratio (OR)=1.98; 95% confidence interval (CI), 1.10–3.56). Out of the 172 *p53* gene exon 4 informative male OSCCs, 72 (41.9%) showed LOH. Among these 72 OSCCs with LOH, the frequency of *Pro* allele loss was 73.6% (53/72). It is notable that alcohol drinking increased the frequency of *Arg* allele loss (OR=10.56; 95% CI, 1.23–234.94) in OSCCs from patients who both smoked cigarettes and chewed areca quid (AQ). The frequency of LOH of *p53* was not different between *p53*-mutated OSCCs and *p53*-normal OSCCs. Thus, the present study revealed that (a) the *Arg* allele is associated with *p53* mutations, (b) the *Pro* allele is preferentially lost in OSCCs associated with cigarette smoking and AQ chewing, while the frequency of *Arg* allele loss is increased with alcohol drinking, and (c) haploinsufficiency of *p53* is in itself likely to contribute to tumour progression in Taiwanese OSCCs.

Oral cancer is the sixth most common cancer in the world (Nair *et al*, 1996), and was accounted to be the fifth leading cause of male cancer mortality in Taiwan in 2002 (Department of Health, ROC, 2003). Epidemiological studies have clearly indicated that areca quid (AQ) chewing, cigarette smoking and alcohol are the major risk factors for oral squamous cell carcinoma (OSCC) (IARC, 1985; Ko *et al*, 1995). In Taiwan, approximately 80% of all oral cancer patients are associated with the AQ chewing habit (Ko *et al*, 1995). In addition, most Taiwanese AQ chewers are also smokers and alcohol drinkers.

The p53 tumour suppressor protein is involved in cell-cycle control, apoptosis and DNA repair (Vogelstein *et al*, 2000). The importance of the *p53* tumour suppressor gene in the process of carcinogenesis is well established (Hussain and Harris, 1998). Mutation at *p53* has been demonstrated in over 50% of human cancers, especially tobacco-related cancers. Recently, we reported an important contributive role for tobacco carcinogens in *p53* mutation for a series of Taiwanese patients with OSCCs (Hsieh *et al*, 2001). The most prevalent types of *p53* mutation found in Taiwanese OSCCs were G:C to A:T transitions and G:C to T:A transversions. Studies have demonstrated that these types of mutations are the most common mutations observed in animals treated with NNK or other tobacco nitrosamines (Belinsky *et al*, 1991; Oreffo *et al*, 1993; Ronai *et al*, 1993; Chang *et al*, 1996). Evidence from the literature also indicates that NNK-associated DNA adducts, in addition to being repaired by the nucleotide excision repair pathway, are also repaired by base excision repair (Cloutier and Castonguay, 1998). *XRCC1* plays an important role in the base excision repair pathway, and interacts with DNA polymerase *β*, PARP and DNA ligase III. It also has a BRCT domain, which is characteristic of proteins involved in cycle checkpoint functions and this domain may be responsive to DNA damage (Caldecott *et al*, 1996; Masson *et al*, 1998). Abdel-Rahman and El-Zein (2000) found that the 399*Gln* polymorphism of *XRCC1* appeared to be associated with reduced repair of NNK-induced genetic damage in cultured human lymphocytes. We found that OSCC patients with an *XRCC1* 399*Gln*/*Gln* genotype exhibited a significantly higher frequency of *p53* mutation than those with an *Arg*/*Gln* or an *Arg*/*Arg* genotype (Hsieh *et al*, 2003).

It is known that one type of *p53* polymorphism that is found in the general population results in either an Arg or a Pro at residue 72 and this produces a marked change in the structure of the p53 protein (Matlashewski *et al*, 1987). Recently, Marin *et al* (2000) and Tada *et al* (2001) have reported that the *Arg72* allele was preferentially mutated and retained in various human tumours. In addition to codon 72 polymorphism, Wu *et al* (2002) found that cells with different *p53* haplotypes of exon 4–intron 3–intron 6 showed different capacities for DNA repair and apoptosis. In this study, we test whether different *p53* haplotypes of exon 4–intron 3–intron 6 would associate with *p53* mutations and examine the role of the *Arg72* allele in loss of heterozygosity (LOH) of *p53* gene in Taiwanese OSCCs.

## MATERIALS AND METHODS

### Patients, tissue specimens and clinical diagnosis

This study was approved by the Institutional Review Board, Chang Gung Memorial Hospital. A total of 715 OSCC patients treated at Chang Gung Memorial Hospital, Lin-Kuo during the period from March 1999 to December 2001 were recruited consecutively for participation in the present study. All cases were histologically confirmed. Female patients (*n*=42) were excluded from this study because of an insufficient number. Those who were diagnosed as nonsquamous cell carcinoma (*n*=44) were also excluded. Thus, a total of 629 male OSCC patients, including 237 patients previously studied (Hsieh *et al*, 2003), were included for the present analysis. All patients gave informed consent for participation and were interviewed uniformly before surgery by a well-trained interviewer. The questionnaire used in the interview sought detailed information on current and past cigarette smoking, alcohol drinking and AQ chewing habits, occupational history, family disease history, dietary history and general demographic data.

For each case, two samples were taken, a tumour sample and a sample from the adjacent normal nontumour tissue. These samples were surgically dissected into small pieces, frozen immediately in liquid nitrogen and stored at −80°C. In addition, 10 ml of venous blood was drawn into heparinised tubes (Vacutainer) and stored at 4°C. The whole blood was separated into plasma, buffy coat cells and red blood cells by centrifugation within 18 h of obtaining the blood, and stored in a −80°C freezer. Genomic DNA for genotyping was extracted and purified from the buffy coat cells as previously described (Hsieh *et al*, 2003).

The surgically removed samples were sent to the Department of Pathology, Chang Gung Medical Center for examination and scored according to the recommendations for the reporting of specimens containing oral cavity and oropharynx neoplasms by the Associations of Directors of Anatomic and Surgical Pathology (ADASP) (2000). Histology diagnosis was defined as squamous cell carcinoma, verrucous carcinoma, cylindric cell carcinoma, adenoid cystic carcinoma, mucoepidermoid carcinoma and adenocarcinoma.

As reference male controls, 371 subjects with available blood samples selected from 3000 random samples of the Taiwanese general population collected to study blood lead concentrations were included in this study (Hsieh *et al*, 2000).

### Tobacco, AQ and alcohol use

Study participants were asked if they had ever smoked cigarettes, chewed AQ or drank alcohol on a regular basis (at least once a week for 1 year). Those who responded ‘yes’ to these questions were classified as tobacco, AQ and alcohol users, respectively.

### *p53* genotype and haplotype determination

The genotype of *p53* for intron 3, exon 4 and intron 6 was determined by PCR-RFLP (Wu *et al*, 2002) followed by polyacrylamide gel electrophoresis. For the exon 4 codon 72 polymorphism, a 396 bp DNA fragment was amplified and digested with *Bst*UI. The *Arg/Arg* homozygotes showed two bands of 165 and 231 bp, the *Arg/Pro* heterozygotes showed three bands of 165, 231 and 396 bp and the *Pro/Pro* homozygotes showed only one band of 396 bp. For the intron 3 polymorphism, either a 180 or 196 bp DNA fragment was amplified. The WW (without the 16 bp duplication) homozygotes showed one band of 180 bp, the WM heterozygotes showed two bands of 180 and 196 bp and the MM (with the 16 bp duplication) homozygotes showed only one band of 196 bp. For the intron 6 polymorphism, a 404 bp DNA fragment was amplified and digested with *Msp*I. The GG homozygotes showed two bands of 68 and 336 bp, the GA heterozygotes showed three bands of 68, 336 and 404 bp and the AA homozygotes showed only one band of 404 bp.

*p53* haplotypes could be inferred directly from the genotyping results for the individuals who were heterozygous at only one site or at no sites. For the other individuals, allele cloning and PCR-RFLP methods were used to determine the haplotypes. A 1.6 kb fragment of *p53* gene containing intron 3, exon 4 and intron 6 was amplified, cloned with the pCR® 2.1 vector (Invitrogen, Carlsbad, CA, USA) and transformed into INVαF′ cells. At least five white clones of each case were genotyped for intron 3, exon 4 and intron 6 by PCR-RFLP as described above.

### Mutation and LOH analysis of the *p53* gene

According to Soussi and Beroud (2001), 13.6% of *p53* mutations were located outside exons 5–8, with a significant number of mutations in exons 4, 10 and, to a lesser extent, 9. Therefore, single-stranded conformation polymorphism (SSCP) analysis was used to analyse tumour samples for mutations within exons 4–10 of the *p53* gene. The lengths of PCR fragments for our SSCP analysis were in the range of 135–245 bp and the sensitivity and specificity of this technique to detect mutations even if only present in a low amount is more than 90%. Cases displaying an altered electrophoretic mobility were reamplified in another reaction and analysed by direct sequencing of both strands to confirm and characterise the nature of the mutation (Hsieh *et al*, 2001).

**Figure 1:**
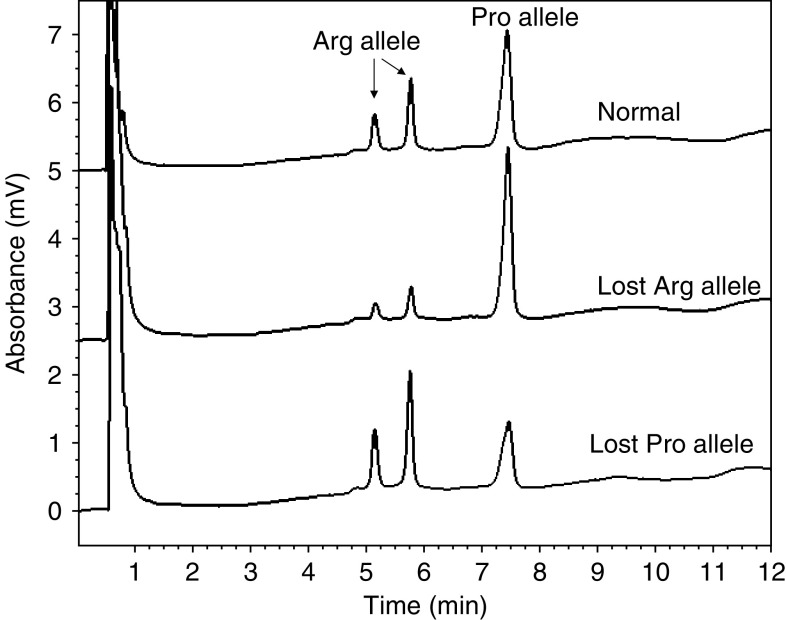
LOH analysis of *p53* gene by DHPLC. The analysis was performed at 50°C and flow rate 0.9 ml min^−1^ in a gradient of acetonitrile in 0.1 M triethylammonium acetate: the gradient started at 8.75%, increased in 3.5 min to 13.75%, then increased in 7 min to 16.25%, was constant for 1 min, then increased in 1 min to 75%, was constant for 1 min (wash), then decreased in 1 min to 8.75% and finally was constant for 1 min (equilibration).

For individuals who were heterozygous at exon 4 codon 72, DNA from their tumour tissues was used to amplify the 396 bp exon 4 fragment, which was digested with *Bst*UI, purified and analysed by denaturing high-performance liquid chromatography (DHPLC) (WAVE DNA Fragment Analysis System, Omaha, NE, USA) (Kleymenova and Walker, 2001). PCR was performed for 27 cycles so that the reaction was still in the exponential phase of amplification. Then, DHPLC analysis was performed at 50°C and flow rate 0.9 ml min^−1^ in a gradient of acetonitrile in 0.1 M triethylammonium acetate: the gradient started at 8.75%, increased in 3.5 min to 13.75%, then increased in 7 min to 16.25%, was constant for 1 min, then increased in 1 min to 75%, was constant for 1 min (wash), then decreased in 1 min to 8.75% and finally was constant for 1 min (equilibration). The ratio of the peak area between the 396 bp fragment, the Pro allele, and the 231 bp fragment, the Arg allele, was calculated. Tumour DNA with a ratio outside the 95% confidence interval (CI) of the mean ratio from 30 normal controls was considered as positive for LOH in the *p53* gene (representative results by DHPLC are shown in Figure 1).

## STATISTICAL ANALYSIS

Statistical analyses were performed with the Statistical Analysis System (SAS) version 8.1. The association between the frequency of *p53* mutation or LOH and genotype/haplotype of *p53* intron 3, exon 4 and intron 6 was examined by *χ*^2^ or Fisher's exact test. Multiple logistic regression model was further used to assess the major factors attributed to the frequency of *p53* mutation.

## RESULTS

A total of 629 consecutive patients with a diagnosis of OSCC were enrolled in the study. The demographic information of the patients is shown in Table 1. The most common primary sites were the bucca and the tongue. In all, 94% (591/628) of the patients had smoked at some time, 62.4% (389/623) were users of alcohol at some time and 90.8% (570/628) had chewed AQ at some time.

As shown in Table 2, the frequencies of *Arg*72, W allele of intron 3 and G allele of intron 6 were 0.58, 0.97 and 0.97, respectively, in the referent male controls. The frequencies of the *Arg/Arg*, *Arg/Pro* and *Pro/Pro* genotypes for codon 72 were 34.50, 47.71 and 17.79%, respectively. The frequencies of the WW, WM and MM genotypes for intron 3 were 94.07, 5.39 and 0.54%, respectively. The frequencies of the GG, GA and AA genotypes for intron 6 were 94.07, 5.39 and 0.54%, respectively. All of the distributions were in Hardy–Weinberg equilibrium. The most common two haplotypes of exon 4–intron 3–intron 6 were Arg-W-G (58.36%), followed by Pro-W-G (38.41%). All other types were relatively rare. It is interesting to note that these rare haplotypes were slightly associated with a risk of oral cancer (odds ratio (OR)=1.72; 95% CI, 0.97–3.07).

Tumour samples from 272 OSCC patients without antecedent treatment, including 237 cases published previously (Hsieh *et al*, 2001), were examined for mutations within exons 4–10 of the *p53* gene by PCR-SSCP. Based on Table 2, the associations between the common haplotypes of *p53* gene and cancer risk were slightly different in different sites of oral cancer (oral cavity cancer *vs* hypopharyngeal/oropharyngeal cancer). The cases of hypopharyngeal and oropharyngeal cancer were excluded for statistical analysis (*n*=18). Of the 254 oral cavity OSCCs, 128 (50.4%) showed a *p53* gene mutation at exons 4–10. Individuals with either one or no Pro-W-G alleles were more likely to have *p53* gene mutations (OR=1.99; 95% CI, 1.08–3.68; Table 3) than those with two Pro-W-G alleles. After adjustment for age, cigarette smoking, alcohol drinking, AQ chewing and *XRCC1* 399*Gln* polymorphism, individuals with one or no Pro-W-G alleles still had a higher frequency of *p53* mutations (OR=1.98; 95% CI, 1.10–3.56) than those with two Pro-W-G alleles.

Among the 128 OSCCs with *p53* mutations by SSCP, 12 samples contained two different mutations of the *p53* gene and 15 samples were not successfully sequenced. To reduce the complexity of interpretation, cases with two mutations were excluded from further analysis. Among the 101 mutations with an identified sequence, 16 (15.8%) were deletions or insertions, 53 (52.5%) were G:C to A:T transitions and 17 (16.8%) were G:C to T:A transversions. The forms of mutation were not significantly different between individuals with two Pro-W-G alleles and those with either one or no Pro-W-G alleles (data not shown).

Tumour samples from 172 informative male oral cavity cancer patients for *p53* gene exon 4 were examined for LOH of the *p53* gene. Of the 172 OSCCs, 72 (41.9%) showed LOH (Table 4). Among the 72 OSCCs with LOH of the *p53* gene, Pro allele loss was found in 53 (73.6%) of the tumours. The frequency of LOH of *p53* was not associated with age, cigarette smoking, AQ chewing or alcohol drinking. However, stage IV tumours had a slightly higher frequency (33/66, 50.0%) of *p53* LOH than stage I–III tumours (39/106, 36.8%) (*P*=0.09 by *χ*^2^). It is also interesting to note that alcohol drinking would seem to increase the frequency of *Arg* allele loss (OR=10.56; 95% CI, 1.23–234.94) among cigarette smokers and AQ chewers.

The status of *p53* gene mutation was known for 94 tumour samples from 172 informative male oral cavity cancer patients for *p53* gene exon 4. Of the 94 OSCCs, 67 (71.3%) showed alterations (mutation, LOH or both) of *p53* gene (Table 5). The frequency of LOH of *p53* was not different between 46 *p53*-mutated OSCCs and 48 *p53*-normal OSCCs (52.2 *vs* 43.8%, *P*=0.41 by *χ*^2^). However, LOH was more likely to appear at late than early tumour stage (14 events for stage IV *vs* seven events for stage I–III), while mutation occurred at the early tumour stage (16 events for stage I–III *vs* six events for stage IV). Therefore, stage IV tumours had a slightly higher frequency (26/45, 57.8%) of *p53* LOH than stage I–III tumours (19/49, 38.8%) (*P*=0.07 by *χ*^2^). Furthermore, a significant cumulative effect of mutation of *p53* and tumour stage on LOH of *p53* was observed (*P*=0.03 by *χ*^2^ trend test). It is also interesting to note that stage IV tumours had a slightly higher frequency (20/26, 76.9%) of *Pro* allele loss than stage I–III tumours (11/19, 57.9%) (*P*=0.17 by *χ*^2^) regardless of *p53* mutation. The frequency of *p53* mutation was not different between 31 OSCCs having *Pro* allele loss and 14 OSCCs with *Arg* allele loss (51.6 *vs* 57.1%, *P*=0.73 by *χ*^2^).

## DISCUSSION

p53 has a critical role in cell-cycle control, apoptosis and DNA repair (Vogelstein *et al*, 2000). Among the more than 10 DNA sequence polymorphisms identified, the codon 72 polymorphism (Arg/Pro) has been explored in depth for a potential association with cancer. Although this polymorphism has been implied to be associated with certain cancer types (Yu *et al*, 1999; Fan *et al*, 2000; Shepherd *et al*, 2000), Sun *et al* (1999) reviewed 31 epidemiological case–control studies and suggested that codon 72 allelism did not have an impact on human cancer risk. The present study found that there was no significant difference in the frequency of codon 72 genotype between patients and referent controls (*P*=0.27). Recently, five case–control studies have been conducted to investigate an association between the *p53* codon 72 polymorphism and the risk of squamous cell carcinoma of the head and neck (SCCHN), but none of them have found a positive association (Hamel *et al*, 2000; McWilliams *et al*, 2000; Summersgill *et al*, 2000; Shen *et al*, 2002; Mitra *et al*, 2003). Taken together, these results suggested that codon 72 allelism does not seem to have an impact on human cancer risk, especially for SCCHN, although Dumont *et al* (2003) had demonstrated that the Arg72 form of p53 was at least five times better at inducing apoptosis than the Pro72 variant.

The difference in overall haplotype frequency between patients and referent controls was not significant in the present study (*P*=0.22). However, it is interesting to note that the rare haplotypes (rare allele in introns 3 and 6) were slightly associated with a risk of oral cancer (OR=1.72; 95% CI, 0.97–3.07) (Table 2). Recently, Gemignani *et al* (2004) have observed that a rare allele in intron 3 was associated with an increased risk of colorectal cancer and reduced basal levels of *p53* mRNA in immortalised lymphoblastoid cell lines. Taken together, these results could support the speculation by Wu *et al* (2002) that introns 3 and 6 rare alleles might exert a functional effect, because as the copy number of the rare allele in introns 3 and 6 is increased, the level of the apoptotic index is decreased.

In Taiwan, most of male oral cavity cancer patients have a history of both habitual cigarette smoking and AQ chewing. In the present study, among 167 male OSCC patients informative for *p53* gene exon 4, 141 (84.4%) have a history of both habitual cigarette smoking and AQ chewing. Among these 141 OSCC patients, 91 (64.5%) patients also had a history of habitual alcohol drinking. The frequency of LOH for *p53* did not differ between patients with and without alcohol drinking. However, interestingly, almost all of the patients (95.5%, 21/22) without alcohol drinking had lost the *Pro* allele, whereas only 68.4% (26/38) of the patients with alcohol drinking had lost the *Pro* allele. In this study, we did not determine which allele was mutated in the *p53*-mutated oral cavity cancer patients with germline heterozygosity (*Arg/Pro*). Further studies to determine the biological mechanism by which there is selection for different codon 72 alleles by alcohol drinking are thus desirable.

In the present study, we found that OSCC patients with the *Arg*72 allele had a significantly higher frequency of *p53* mutation than those with *Pro/Pro* genotype among patients with common alleles of introns 3 and 6 (OR=1.93; 95% CI, 1.03–3.63). In addition, 73.6% (53/72) of those who showed LOH of the *p53* gene had lost the *Pro* allele. These results are consistent with those reported by other authors for some tumour types, in which it has been pointed out that LOH of *p53* occurs more commonly for the *Pro* allele and that there is a preferential mutation of the *Arg*72 allele (Brooks *et al*, 2000; Marin *et al*, 2000; Tada *et al*, 2001; Furihata *et al*, 2002; Anzola *et al*, 2003). Based on the reports assigning a higher apoptotic potential to the *Arg* allele (Wu *et al*, 2002) and the current data, we could hypothesise that the cells carrying an *Arg* allele require further *p53* mutations to increase their tumorigenic potential, while *Pro/Pro* cells could undergo this procedure with fewer damage. Indeed, Marin *et al* (2000) and Tada *et al* (2001) found that the p53 mutant acts as a more potent inhibitor of p73 activity when p53 has *Arg*72 rather than *Pro*72. Additional comprehensive studies using this series of samples will be needed to elucidate the association between polymorphisms within *p53* and the mutant behaviour of *p53* in OSCCs associated with cigarette smoking, AQ chewing and alcohol drinking.

The two-hit paradigm of tumour suppressor gene mutation has held sway for many years. Recently, Venkatachalam *et al* (1998) found that a high proportion of the tumours from the *p53*+/− mice retained an intact, functional, wild-type allele. This result indicates that mere reduction in p53 levels may be sufficient to promote tumorigenesis. Although *p53* mutation accompanied by complete loss of the wild-type allele is the most frequent event observed in many human cancers (Greenblatt *et al*, 1994), we found no preference of mutation with LOH (*n*=24), mutation without LOH (*n*=22) or LOH without mutation (*n*=21) in 94 Taiwanese OSCCs with germline heterozygosity in codon 72 (*Arg/Pro*). Furthermore, LOH was more likely to appear at late than early tumour stage (14 for stage IV *vs* seven for stage I–III), while mutation was occurred at the early tumour stage (16 for stage I–III *vs* six for stage IV). These results support the hypothesis that haploinsufficiency of *p53* is in itself likely to contribute to tumour progression and the mutant form of *p53* has a dominant gain-of-function activity or it may block the wild-type protein by acting as a dominant negative (Santarosa and Ashworth, 2004).

In conclusion, we found that *p53* codon 72 polymorphism may not be a dominant factor for predisposition to OSCCs in Taiwan. However, the present study revealed that (a) the Arg allele is associated with *p53* mutations, (b) the Pro allele is preferentially lost in OSCCs associated with cigarette smoking and AQ chewing, while the frequency of Arg allele loss is increased with alcohol drinking, and (c) haploinsufficiency of *p53* is in itself likely to contribute to tumour progression in Taiwanese OSCCs.

## Figures and Tables

**Table 1:** Characteristics of the male patients with OSCCs (*n*=629)

Age (years)	
Mean±s.d.	49.93±10.67
Range	25–78
	
Site of primary tumour (*N* (%))	
Oral cavity	523 (83.2)
Lip	19 (3.0)
Tongue	175 (27.8)
Mouth floor	22 (3.5)
Buccal mucosa	208 (33.1)
Gingiva	53 (8.4)
Hard palate	13 (2.1)
Retromolar trigone	33 (5.2)
Oropharynx	49 (7.8)
Hypopharynx	57 (9.1)
	
Clinical stage (*N* (%))	
Stage I	79 (12.8)
Stage II	136 (22.1)
Stage III	125 (20.3)
Stage IV	275 (44.7)
	
Cigarette smoker at some time (*N* (%))	591 (94.1)
Alcohol drinker at some time (*N* (%))	389 (62.4)
Areca quid chewer at some time (*N* (%))	570 (90.8)

**Table 2:** Risk estimates for *p53* haplotypes in male OSCC patients and control subjects

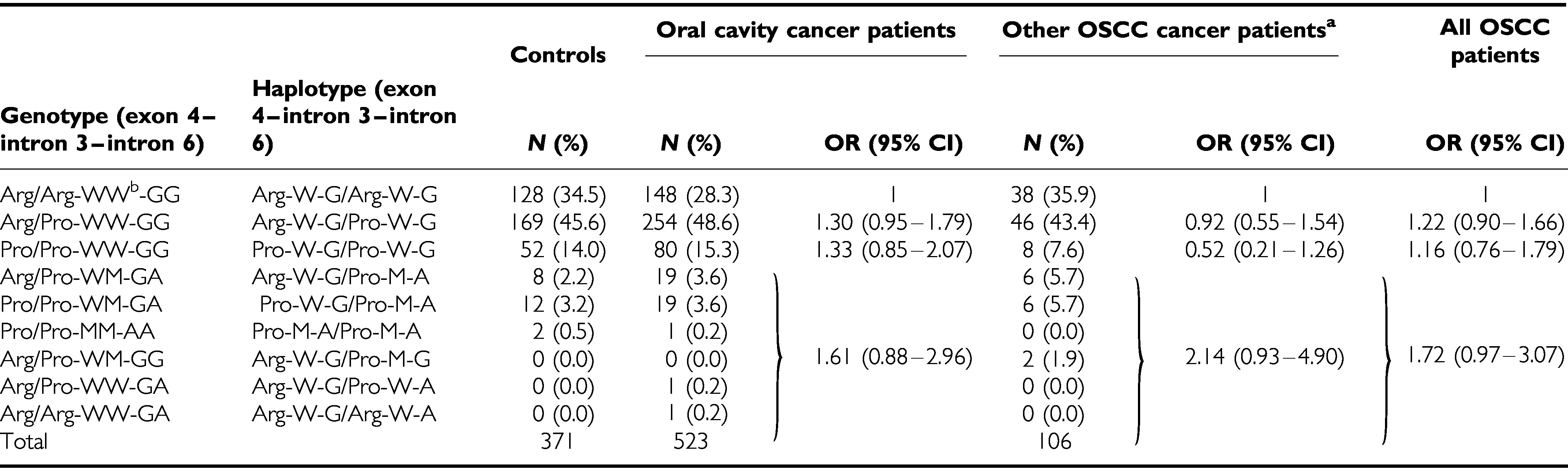

^a^Other OSCC patients: including oropharynx and hypopharynx cancer patients.

^b^W: common allele (without replicative 16 bp); M : rare allele (with replicative 16 bp).

**Table 3:** Association of *p53* haplotypes and *p53* gene mutations in oral cavity cancer

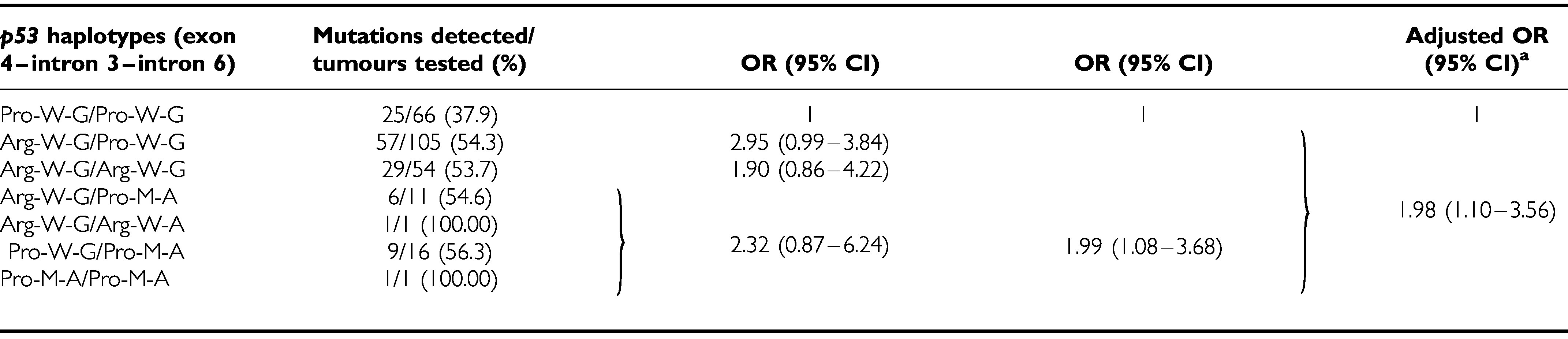

^a^Adjusted for age, cigarette smoking, alcohol drinking, AQ chewing and XRCC1 399Gln polymorphism.

**Table 4:** Stratification analysis of the risk factors for oral cavity cancer and the frequency of LOH of the *p53* gene

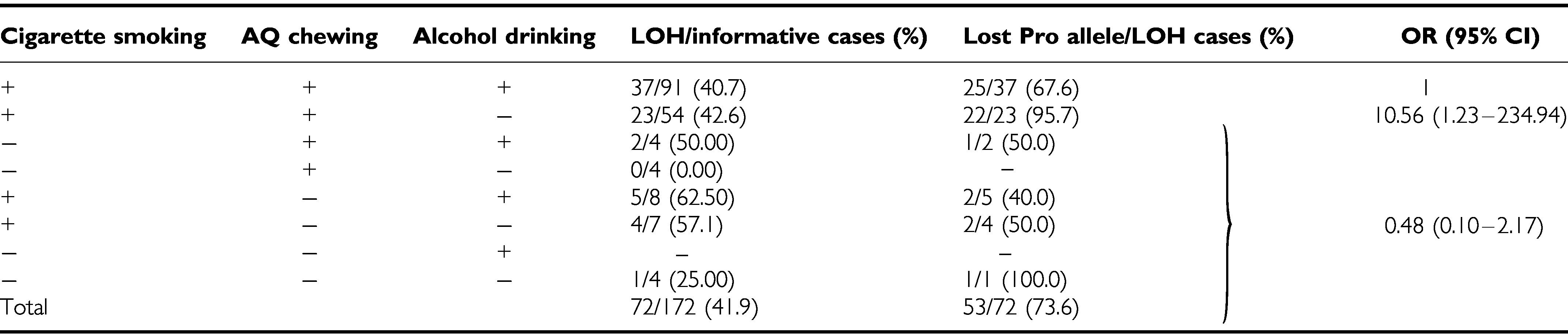

**Table 5:** Association of mutation of *p53*, tumour stage and the frequency of LOH of the *p53* gene in oral cavity cancer

**Mutation of *p53***	**Tumour stage**	**LOH/informative cases (%)**	OR (95% CI)*	**Lost Pro allele/LOH cases (%)**	**OR (95% CI)**
No	I–III	7/21 (33.3)	1	4/7 (57.1)	1
Yes	I–III	12/28 (42.9)	1.50 (0.40–5.75)	7/12 (58.3)	
No	IV	14/27 (51.0)	2.15 (0.57–8.35)	11/14 (78.6)	2.42 (0.56–10.77)
Yes	IV	12/18 (66.7)	4.00 (0.88–19.32)	9/12 (75.0)	
Total		45/94 (47.9)		31/45 (68.9)	

* *P*=0.03 by *χ*^2^ trend test.

## References

[bib1] Abdel-Rahman SZ, El-Zein RA (2000) The 399Gln polymorphism in the DNA repair gene *XRCC1* modulates the genotoxic response induced in human lymphocytes by the tobacco-specific nitrosamine NNK. Cancer Lett 159: 63–71 10974407 10.1016/s0304-3835(00)00532-2

[bib2] Anzola M, Cuevas N, Lopez-Martinez M, Saiz A, Burgos JJ, de Pancorbo MM (2003) Frequent loss of p53 codon 72 Pro variant in hepatitis C virus-positive carriers with hepatocellular carcinoma. Cancer Lett 193: 199–205 12706878 10.1016/s0304-3835(03)00046-6

[bib3] Association of Directors of Anatomic and Surgical Pathology (2000) Recommendations for the reporting of specimens containing oral cavity and oropharynx neoplasms. Mod Pathol 13: 1038–1041 11007046 10.1038/modpathol.3880188

[bib4] Belinsky SA, Devereux TR, White CM, Foley JF, Maronpot RR, Anderson MW (1991) Role of Clara cells and type II cells in the development of pulmonary tumors in rats and mice following exposure to a tobacco-specific nitrosamine. Exp Lung Res 17: 263–278 2050030 10.3109/01902149109064417

[bib5] Brooks LA, Tidy JA, Gusterson B, Hiller L, O’Nions J, Gasco M, Marin MC, Farrell PJ, Kaelin Jr WG, Crook T (2000) Preferential retention of codon 72 arginine p53 in squamous cell carcinomas of the vulva occurs in cancers positive and negative for human papillomavirus. Cancer Res 60: 6875–6877 11156383

[bib6] Caldecott KW, Aoufouchi S, Johnson P, Shall S (1996) *XRCC1* polypeptide interacts with DNA polymerase beta and possibly poly (ADP-ribose) polymerase, and DNA ligase III is a novel molecular ‘nick-sensor’ *in vitro*. Nucleic Acids Res 24: 4387–4394 8948628 10.1093/nar/24.22.4387 PMC146288

[bib7] Chang KW, Lin SC, Koos S, Pather K, Solt D (1996) *p53* and *Ha-ras* mutations in chemically induced hamster buccal pouch carcinomas. Carcinogenesis 17: 595–600 8631150 10.1093/carcin/17.3.595

[bib8] Cloutier JF, Castonguay A (1998) Modulation of DNA repair by various inhibitors of DNA synthesis following 4-(methylnitrosamino)-1-(3-pyridyl)-1-butanone (NNK) induced DNA damage. Chem Biol Interact 110: 7–25 9566722 10.1016/s0009-2797(97)00114-2

[bib9] Department of Health, ROC (2003) Health and Vital Statistics, Republic of China 2002. Taipei, ROC: Department of Health, Executive Yuan

[bib10] Dumont P, Leu JI, Della Pietra III AC, George DL, Murphy M (2003) The codon 72 polymorphic variants of p53 have markedly different apoptotic potential. Nat Genet 33: 357–365 12567188 10.1038/ng1093

[bib11] Fan R, Wu MT, Miller D, Wain JC, Kelsey KT, Wiencke JK, Christiani DC (2000) The p53 codon 72 polymorphism and lung cancer risk. Cancer Epidemiol Biomarkers Prev 9: 1037–1042 11045785

[bib12] Furihata M, Takeuchi T, Matsumoto M, Kurabayashi A, Ohtsuki Y, Terao N, Kuwahara M, Shuin T (2002) p53 mutation arising in Arg72 allele in the tumorigenesis and development of carcinoma of the urinary tract. Clin Cancer Res 8: 1192–1195 12006537

[bib13] Gemignani F, Moreno V, Landi S, Moullan N, Chabrier A, Gutierrez-Enriquez S, Hall J, Guino E, Peinado MA, Capella G, Canzian FA (2004) TP53 polymorphism is associated with increased risk of colorectal cancer and with reduced levels of TP53 mRNA. Oncogene 23: 1954–1956 14647431 10.1038/sj.onc.1207305

[bib14] Greenblatt MS, Bennett WP, Hollstein M, Harris CC (1994) Mutations in the p53 tumor suppressor gene: clues to cancer etiology and molecular pathogenesis. Cancer Res 54: 4855–4878 8069852

[bib15] Hamel N, Black MJ, Ghadirian P, Foulkes WD (2000) No association between p53 codon 72 polymorphism and risk of squamous cell carcinoma of the head and neck. Br J Cancer 82: 757–759 10732740 10.1054/bjoc.1999.0993 PMC2374404

[bib16] Hsieh LL, Chien HT, Chen I H, Liao CT, Wang HM, Jung SM, Wang PF, Chang JT, Chen MC, Cheng AJ (2003) The XRCC1 399Gln polymorphism and the frequency of p53 mutations in Taiwanese oral squamous cell carcinomas. Cancer Epidemiol Biomarkers Prev 12: 439–443 12750239

[bib17] Hsieh LL, Liou SH, Chen YH, Tsai LC, Yang T, Wu TN (2000) Association between aminolevulinate dehydrogenase genotype and blood lead levels in Taiwan. J Occup Environ Med 42: 151–155 10693075 10.1097/00043764-200002000-00009

[bib18] Hsieh LL, Wang PF, Chen IH, Liao CT, Wang HM, Chen MC, Chang JTC, Cheng AJ (2001) Characteristics of mutations in the *p53* gene in oral squamous cell carcinoma associated with betel quid chewing and cigarette smoking in Taiwanese. Carcinogenesis 22: 1497–1503 11532872 10.1093/carcin/22.9.1497

[bib19] Hussain SP, Harris CC (1998) Molecular epidemiology of human cancer: contribution of mutation spectra studies of tumor suppressor genes. Cancer Res 58: 4023–4037 9751603

[bib20] IARC (1985) Betel-quid and Areca Nut Chewing. Lyon: IARC Scientific Publications

[bib21] Kleymenova E, Walker CL (2001) Determination of loss of heterozygosity in frozen and paraffin embedded tumors by denaturing high-performance liquid chromatography (DHPLC). J Biochem Biophys Methods 47: 83–90 11179764 10.1016/s0165-022x(00)00154-8

[bib22] Ko YC, Huang YL, Lee CH, Chen MJ, Lin LM, Tsai CC (1995) Betel quid chewing, cigarette smoking and alcohol consumption related to oral cancer in Taiwan. J Oral Pathol Med 24: 450–453 8600280 10.1111/j.1600-0714.1995.tb01132.x

[bib23] Marin MC, Jost CA, Brooks LA, Irwin MS, O’Nions J, Tidy JA, James N, McGregor JM, Harwood CA, Yulug IG, Vousden KH, Allday MJ, Gusterson B, Ikawa S, Hinds PW, Crook T, Kaelin Jr WG (2000) A common polymorphism acts as an intragenic modifier of mutant p53 behaviour. Nat Genet 25: 47–54 10802655 10.1038/75586

[bib24] Masson M, Niedergang C, Schreiber V, Muller S, Menissier-de Murcia J, de Murcia G (1998) *XRCC1* is specifically associated with poly(ADP-ribose) polymerase and negatively regulates its activity following DNA damage. Mol Cell Biol 18: 3563–3571 9584196 10.1128/mcb.18.6.3563 PMC108937

[bib25] Matlashewski GJ, Tuck S, Pim D, Lamb P, Schneider J, Crawford LV (1987) Primary structure polymorphism at amino acid residue 72 of human p53. Mol Cell Biol 7: 961–963 3547088 10.1128/mcb.7.2.961-963.1987 PMC365159

[bib26] McWilliams JE, Evans AJ, Beer TM, Andersen PE, Cohen JI, Everts EC, Henner WD (2000) Genetic polymorphisms in head and neck cancer risk. Head Neck 22: 609–617 10941163 10.1002/1097-0347(200009)22:6<609::aid-hed10>3.0.co;2-l

[bib27] Mitra S, Chatterjee S, Panda CK, Chaudhuri K, Ray K, Bhattacharyya NP, Sengupta A, Roychoudhury S (2003) Haplotype structure of TP53 locus in Indian population and possible association with head and neck cancer. Ann Hum Genet 67(Part 1): 26–34 12556232 10.1046/j.1469-1809.2003.00005.x

[bib28] Nair J, Ohshima H, Nair UJ, Bartsch H (1996) Endogenous formation of nitrosamines and oxidative DNA-damaging agents in tobacco users. Crit Rev Toxicol 26: 149–161 8688158 10.3109/10408449609017928

[bib29] Oreffo VI, Lin HW, Padmanabhan R, Witschi H (1993) *K-ras* and *p53* point mutations in 4-(methylnitrosamino)-1-(3-pyridyl)-1-butanone-induced hamster lung tumors. Carcinogenesis 14: 451–455 8453721 10.1093/carcin/14.3.451

[bib30] Ronai ZA, Gradia S, Peterson LA, Hecht SS (1993) G to A transitions and G to T transversions in codon 12 of the Ki-ras oncogene isolated from mouse lung tumors induced by 4-(methylnitrosamino)-1-(3-pyridyl)-1-butanone (NNK) and related DNA methylating and pyridyloxobutylating agents. Carcinogenesis 14: 2419–2422 7902220 10.1093/carcin/14.11.2419

[bib31] Santarosa M, Ashworth A (2004) Haploinsufficiency for tumour suppressor genes: when you don’t need to go all the way. Biochim Biophys Acta 1654: 105–122 15172699 10.1016/j.bbcan.2004.01.001

[bib32] Shen H, Zheng Y, Sturgis EM, Spitz MR, Wei Q (2002) p53 codon 72 polymorphism and risk of squamous cell carcinoma of the head and neck: a case–control study. Cancer Lett 183: 123–130 12065086 10.1016/s0304-3835(02)00117-9

[bib33] Shepherd T, Tolbert D, Benedetti J, Macdonald J, Stemmermann G, Wiest J, DeVoe G, Miller MA, Wang J, Noffsinger A, Fenoglio-Preiser C (2000) Alterations in exon 4 of the p53 gene in gastric carcinoma. Gastroenterology 118: 1039–1044 10833478 10.1016/s0016-5085(00)70356-8

[bib34] Soussi T, Beroud C (2001) Assessing *TP53* status in human tumours to evaluate clinical outcome. Nat Rev Cancer 1: 233–240 11902578 10.1038/35106009

[bib35] Summersgill KF, Smith EM, Kirchner HL, Haugen TH, Turek LP (2000) p53 polymorphism, human papillomavirus infection in the oral cavity, and oral cancer. Oral Surg Oral Med Oral Pathol Oral Radiol Endod 90: 334–339 10982955 10.1067/moe.2000.107359

[bib36] Sun Y, Keshava C, Sharp DS, Weston A, McCanlies EC (1999) DNA sequence variants of p53: cancer and aging. Am J Hum Genet 65: 1779–1782 10577934 10.1086/302650 PMC1288390

[bib37] Tada M, Furuuchi K, Kaneda M, Matsumoto J, Takahashi M, Hirai A, Mitsumoto Y, Iggo RD, Moriuchi T (2001) Inactivate the remaining p53 allele or the alternate p73? Preferential selection of the Arg72 polymorphism in cancers with recessive p53 mutants but not transdominant mutants. Carcinogenesis 22: 515–517 11238194 10.1093/carcin/22.3.515

[bib38] Venkatachalam S, Shi YP, Jones SN, Vogel H, Bradley A, Pinkel D, Donehower LA (1998) Retention of wild-type p53 in tumors from p53 heterozygous mice: reduction of p53 dosage can promote cancer formation. EMBO J 17: 4657–4667 9707425 10.1093/emboj/17.16.4657 PMC1170795

[bib39] Vogelstein B, Lane D, Levine AJ (2000) Surfing the p53 network. Nature 408: 307–310 11099028 10.1038/35042675

[bib40] Wu X, Zhao H, Amos CI, Shete S, Makan N, Hong WK, Kadlubar FF, Spitz MR (2002) p53 genotypes and haplotypes associated with lung cancer susceptibility and ethnicity. J Natl Cancer Inst 94: 681–690 11983757 10.1093/jnci/94.9.681

[bib41] Yu MW, Yang SY, Chiu YH, Chiang YC, Liaw YF, Chen CJ (1999) A p53 genetic polymorphism as a modulator of hepatocellular carcinoma risk in relation to chronic liver disease, familial tendency, and cigarette smoking in hepatitis B carriers. Hepatology 29: 697–702 10051470 10.1002/hep.510290330

